# Service-level variation, patient-level factors, and treatment outcome in those seen by child mental health services

**DOI:** 10.1007/s00787-016-0939-x

**Published:** 2017-01-06

**Authors:** Julian Edbrooke-Childs, Amy Macdougall, Daniel Hayes, Jenna Jacob, Miranda Wolpert, Jessica Deighton

**Affiliations:** 1 Evidence Based Practice Unit, Anna Freud Centre, UCL, 21 Maresfield Gardens, London, NW3 5SU UK; 2 Child Outcomes Research Consortium, Anna Freud Centre, UCL, London, UK

**Keywords:** Adolescent mental health, Risk adjustment, Service-level variation, Treatment outcome, Added value score

## Abstract

Service comparison is a policy priority but is not without controversy. This paper aims to investigate the amount of service-level variation in outcomes in child mental health, whether it differed when examining outcomes unadjusted vs. adjusted for expected change over time, and which patient-level characteristics were associated with the difference observed between services. Multilevel regressions were used on *N* = 3256 young people (53% male, mean age 11.33 years) from 13 child mental health services. Outcome was measured using the parent-reported Strengths and Difficulties Questionnaire. The results showed there was 4–5% service-level variation in outcomes. Findings were broadly consistent across unadjusted vs. adjusted outcomes. Young people with autism or infrequent case characteristics (e.g., substance misuse) had greater risk of poor outcomes. Comparison of services with high proportions of young people with autism or infrequent case characteristics requiring specialist input needs particular caution as these young people may be at greater risk of poor outcomes.

## Introduction

Service comparison of treatment outcome is a policy priority across healthcare [1]. Aims include transparency and accountability regarding the impact and quality of service provision in addition to informing service benchmarking and funding. It is not without controversy in terms of defining and interpreting service-level variation [2]. We recommend caution when comparing service-level^1^ outcomes regarding child mental health and well-being, as seemingly objective biological measures of treatment outcome are not applicable. Here, proxy indicators are relied upon from multiple subjective reports [3, 4]. One widely used outcome measure in child mental health services is the Strengths and Difficulties Questionnaire (SDQ, 5) typically reported by parents. However, there are limitations to examining treatment outcome in routine practice because inferences about causation should not be made. Scores may be expected to reduce in the short term without intervention because of regression to the mean [6], attenuation, and normal fluctuation. In an attempt to overcome these limitations, the added value score was developed to adjust for expected change in the parent-reported SDQ had the child not received mental health interventions [7]. Nevertheless, some presenting problems at the outset of treatment may be less likely to demonstrate change on standardised outcome measures, such as autism [8]. Evidence is still needed to inform how to adjust for differences in expected treatment outcomes between services with different patient populations, above and beyond adjusting for expected change within individual patients over time. Risk adjustment is arguably more established in physical health settings [9] but there is a lack of evidence from child mental health services in the UK. Research from outside the UK has demonstrated that baseline symptoms is the only factor consistently associated with treatment outcome in child mental health settings, although a range of other characteristics have also shown significant associations [10–14]. An unpublished thesis examining a sample of 297 service attenders in the UK found that baseline symptoms and case complexity (e.g., problem duration, comorbidity, chronic physical illness) were associated with worse treatment outcomes [15]. Still, service-level variation in treatment outcome was not examined. Recently, evidence from UK child mental health services has shown service-level variation in the allocation of resources, in terms of number of appointments attended, for young people with similar problems and that clusters of problem type and degree of impairment (identified by best practice guidelines and clinical recommendations) were associated with resource utilisation [16]. Correspondingly, it is important to investigate whether there is similar service-level variation in treatment outcome.

The aim of the present research was to investigate (1) the amount of service-level variation in treatment outcome in child mental health, (2) whether it differed when examining outcomes unadjusted vs. adjusted for expected change over time, and (3) which patient-level characteristics were associated with the difference observed between services. In line with the above evidence, we expected moderate amounts of service-level variation in both unadjusted and adjusted treatment outcome with indicators of case complexity being associated with the difference observed between services.

## Method

### Participants and procedure

Data were derived from a routinely collected child mental health dataset that we have described elsewhere [17]. Young people from this dataset were included if their data were reported in or after 2009, their case was closed, and they had complete demographic characteristics and variables of interest at baseline (see the “Measures” section). This resulted in a sample of *N* = 19,275 young people.^2^ In order to examine treatment outcome, change in mental health difficulties from time 1 (T1; assessment) to time 2 (T2; approximately 4–8 months after first assessment) was needed. Therefore, young people from this sample with complete measures of mental health difficulties at T2 were included. This resulted in a sample of *N* = 3739 young people from 32 services in the UK. However, estimations of treatment outcome in services with data from fewer than 100 young people may be unreliable. Therefore, only services with complete data on at least 100 young people were included, which resulted in a final sample of *N* = 3256 young people from 13 services with data from between 110 and 526 children per service; demographic characteristics are presented in Table 1.

**Table 1:** Demographic, case and severity characteristics

	T1 complete only	T1–T2 complete
*N*	16,019	3,256
Gender (male, *n*)^a^	53% (8440)	52% (1679)
Gender (female, *n*)	47% (7565)	49% (1575)
Age, *M* (SD)	11.64 (3.39)***	11.33 (3.42)
White	64% (10,276)***	69% (2247)
Mixed	5% (744)	5% (146)
Asian	8% (1250)***	5% (153)
Black	6% (929)	5% (173)
Other	5% (798)	5% (149)
Not stated or missing ethnicity	13% (2022)	12% (388)
Hyperactivity	9% (1478)	10% (316)
Emotional problems	48% (7723)***	55% (1791)
Conduct problems	20% (3218)	20% (649)
Eating disorder	3% (496)***	5% (156)
Self-harm	8% (1316)	8% (266)
Autism	9% (1396)	9% (296)
Other problems	14% (2258)***	22% (718)
SEN	8% (1217)	8% (268)
Infrequent characteristics^b^	12% (1856)	12% (386)
T1 total difficulties, *M* (SD)	18.78 (7.40)*	18.49 (7.13)
T1 hyperactivity, *M* (SD)	5.90 (2.92)**	5.74 (2.90)
T1 conduct, *M* (SD)	3.97 (2.61)***	3.66 (2.55)
T1 peer problems, *M* (SD)	3.61 (2.46)	3.59 (2.45)
T1 prosocial, *M* (SD)	6.64 (2.50)*	6.75 (2.51)
T1 emotional, *M* (SD)	5.30 (2.80)***	5.49 (2.78)
T1 total impact, *M* (SD)	3.97 (2.94)**	4.13 (2.88)

*SEN* special educational needs, *T1* time 1, *T2* time 2

^a^Gender was dummy coded as there were three response options: 0 = unspecified, 1 = male, 2 = female

^b^Case characteristic occurring with a frequency of <5% were grouped into “infrequent characteristics” to avoid including under-powered groups in the main analysis (also see “Measures”)

* *p* < .05

** *p* < .01

*** *p* < .001

There were a number of significant differences between the wider sample with T1 data only and the included sample with both T1 and T2 data. However, when inspecting the magnitude of these differences, the two samples appear to be broadly comparable. The largest difference was that the included sample had an 8% greater proportion of young people with ‘other presenting problems’ recorded. Therefore, a complete-cases analysis, which is widely used in cohort studies [18] was deemed appropriate.

According to the CORC protocol, questionnaires are completed by young people, parents, and/or clinicians at assessment (T1) and again approximately 6 months later or, if sooner, case closure (T2) [19]. The measures were taken from a secondary analysis of routinely collected data so ethical review was not relevant; the patient and service identifiers were also further anonymised in the present dataset for research purposes [20].

### Measures

#### Demographic characteristics

Age, gender, and ethnicity were recorded by clinicians as part of routine data recording. Ethnicity was captured using the categories from the 2001 Census. These were grouped for analysis as follows: White (including White British, Irish and Other White background), Mixed (including Mixed White and Black Caribbean, Mixed White and Black African, Mixed White and Asian, and any other mixed background), Asian (including Indian, Pakistani, Bangladeshi and Other Asian), Black or Black British (including Caribbean, African and Other Black), and other ethnic groups (including Chinese and Other).

#### Case characteristics

The presence or absence of case characteristics was obtained by clinicians on first contact, including: SEN, hyperactivity, emotional problems, conduct problems, eating disorder, psychosis, self-harm, autism, intellectual disability (also known as learning difficulty in UK health services), developmental disorder, habit disorder, substance misuse, other presenting problems, child protection concerns, and Child Act order in place. Case characteristics occurring with a frequency of <5% were grouped into an ‘infrequent characteristics’ variable to avoid including under-powered groups in the main analysis; i.e., psychosis, intellectual disability, developmental disorder, habit disorder, substance misuse, child protection concerns, and Child Act.

#### Severity characteristics

To measure severity characteristics, the nine-item impact supplement of the Strengths and Difficulties Questionnaire [5, 21] was used. It measures severity, including duration, overall distress and the impairment of mental health difficulties on home life, friendships, classroom performance, and leisure activities. Parents responded to the problem duration items from ‘less than a month’ (0) to ‘over a year’ (3), and to the distress and impairment items from ‘not at all’ (0) to ‘a great deal’ (2).

#### Unadjusted treatment outcome

To measure unadjusted treatment outcome, the 25-item SDQ [5, 21] was used. The SDQ measures mental health symptoms and consists of four subscales assessing difficulties (i.e., conduct problems, emotional problems, peer problems, and hyperactivity) and one assessing strengths (i.e., prosocial). The five subscales can also be summed to create a ‘total difficulties’ score. Parents responded to the items from ‘not true’ (0) to ‘certainly true’ (2). The SDQ is a widely used measure of mental health difficulties; in particular, the internal consistency of the SDQ has been reported as 0.82 [22]. Unadjusted treatment outcome was computed by regressing T2 total difficulties on T1 total difficulties and saving the standardised residual.

#### Adjusted treatment outcome

We examined the added value score in order to examine whether there was a different pattern of associations between patient-level factors and treatment outcome—when also accounting for expected change in treatment outcome, had young people not accessed services. The added value score is the difference between observed and expected change in mental health difficulties from T1 to T2 in a clinical sample and is expressed as an effect size [7, 23]. A score not significantly different from 0 suggests that young people’s mental health difficulties changed no more than would have been expected had they not received mental health interventions: a positive score suggests they improved more than expected, and a negative score suggests they deteriorated more than expected. It was calculated using the following equation:

$$ ( 2. 3 + \left( {0. 8\times {\text{total difficulties at T1}}} \right) + \left( {0. 2\times {\text{total impact at T1}}} \right) - \left( {0. 3\times {\text{emotional difficulties at T1}}} \right) - {\text{total difficulties at T2}})/ 5. $$

### Analytic strategy

To investigate the amount of service-level variation in treatment outcome, multilevel modelling was performed in STATA 12 [24]. Two null models without predictors were computed with unadjusted and adjusted treatment outcome as the criterion variables, and the intraclass correlation coefficient (ICC) was calculated.

To examine whether service-level variation differed when accounting for expected change over time, two criterion variables were used: unadjusted versus adjusted treatment outcome.

To examine whether service-level variation in treatment outcome was explained by patient-level demographic, case and severity characteristics, four random intercept models were tested. The same effects were examined for both criterion variables. In Model 1, the association between demographic characteristics and treatment outcome was examined, and the eight patient-level demographic characteristics were entered as level-1 predictors: male (coded 1 for male); female (coded 1 for female); grand mean centred age; and White, Asian, Mixed, Black, and Other (each dummy coded 1, with not stated as the reference category). In Model 2, the association between case characteristics and treatment outcome was examined, and the nine patient-level case characteristics were entered as level-1 predictors: emotional disorder, self-harm, conduct disorder, eating disorder, hyperactivity, autism, other problems, infrequent characteristics, and special educational needs (SEN) (each coded 1 for present). In Model 3, the association between severity characteristics and treatment outcome was examined, and the 14 patient-level severity characteristics were entered as level-1 predictors: problem duration less than 1 month, between 1 and 5 months, between 6 and 12 months, and missing (each dummy coded 1, with duration more than 1 year as the reference category); and the indicators of distress or impairment caused by mental health difficulties (i.e., overall distress; the impairment of mental health difficulties on home life, friendships, classroom performance, and leisure activities) were each recoded into two dummy coded variables—medium and high severity—with little severity or missing as the reference category. The likelihood ratio test was used to compare the fit of subsequent models, and the amount of service-level variance explained in each of the models described above was examined.

## Results

Regarding the amount of service-level variation in treatment outcome, in the null models, 4–5% of the variance in unadjusted and adjusted treatment outcome was explained at the service-level and 95–96% was residual or unexplained variance. There was a relatively small amount of service-level variation compared to residual or unexplained variation.

Regarding whether service-level variation differed when examining outcomes unadjusted versus adjusted for expected change over time, Tables 2 and 3 show the results of the multilevel regressions predicting unadjusted and adjusted treatment outcome, respectively. The amount of service-level variation did not differ substantially between unadjusted versus adjusted treatment outcomes or across models, ranging between 0.05 and 0.07.

**Table 2:** Multilevel regressions with demographic, case and severity characteristics predicting unadjusted treatment outcome

Parameter estimates	Model 0	Model 1	Model 2	Model 3
	Estimate (SE)	Estimate (SE)	Estimate (SE)	Estimate (SE)
Fixed effects				
Intercept	–0.08 (0.06)	–0.15 (0.69)	0.29 (0.69)	–0.29 (0.69)
Female		0.06 (0.69)	0.18 (0.68)	0.25 (0.68)
Male		0.18 (0.69)	0.22 (0.68)	0.27 (0.68)
Age		–0.03 (0.01)***	–0.02 (0.01)***	–0.02 (0.01)***
White		–0.07 (0.06)	–0.06 (0.06)	–0.06 (0.06)
Mixed		–0.08 (0.10)	–0.07 (0.10)	–0.07 (0.10)
Asian		–0.08 (0.10)	–0.08 (0.10)	–0.07 (0.10)
Black		–0.02 (0.10)	–0.19 (0.09)*	–0.20 (0.09)*
Other			–0.02 (0.10)	–0.02 (0.10)
Hyperactivity			0.19 (0.06)**	0.18 (0.06)**
Emotional problems			–0.08 (0.04)	–0.08 (0.04)
Conduct problems			0.09 (0.05)	0.09 (0.05)
Parameter estimates				
Eating disorder			–0.17 (0.08)*	–0.13 (0.08)
Self-harm			0.02 (0.07)	0.02 (0.07)
Autism			0.34 (0.06)***	0.32 (0.06)***
Other problems			0.08 (0.05)	0.08 (0.05)
SEN			0.14 (0.07)*	0.13 (0.07)
Low-frequency case characteristics			0.12 (0.05)*	0.12 (0.05)*
<1 month duration vs. >1 year				0.23 (0.21)
1- to 5-month duration vs. >1 year				–0.28 (0.09)**
6- to 12-month duration vs. >1 year				–0.11 (0.07)
Problem duration missing vs. >1 year				–0.03 (0.07)
Moderate distress				–0.03 (0.06)
Parameter estimates				
High distress				–0.03 (0.06)
Moderate home-life impairment				–0.09 (0.05)
High home-life impairment				–0.06 (0.06)
Moderate friendships impairment				0.01 (0.05)
High friendships impairment				0.05 (0.07)
Moderate classroom impairment				–0.07 (0.06)
High classroom impairment				0.07 (0.06)
Moderate leisure impairment				0.08 (0.06)
High leisure impairment				–0.04 (0.07)
Variance components				
Residual variance	0.96 (0.01)	0.94 (0.01)	0.63 (0.01)	0.90 (0.01)
Service-level variance	0.05 (0.05)	0.06 (0.05)	0.06 (0.05)	0.05 (0.05)

*N* = 3256. Findings in bold indicate that the pattern (in terms of improvement of model fit and significance of the coefficient) was the same as in the multilevel regressions predicting adjusted treatment outcome (also see Table 3)

*SEN* special educational needs

* *p* < .05

** *p* < .01

*** *p* < .001

**Table 3:** Multilevel regressions with demographic, case and severity characteristics predicting adjusted treatment outcome

Parameter estimates	Model 0	Model 1	Model 2	Model 3
	Estimate (SE)	Estimate (SE)	Estimate (SE)	Estimate (SE)
Fixed effects				
Intercept	0.25 (0.07)***	0.29 (0.85)	0.27 (0.09)**	0.09 (0.09)
Female		–0.10 (0.84)		
Male		–0.13 (0.84)		
Age		0.03 (0.01)***		
White		0.09 (0.07)		
Mixed		0.09 (0.12)		
Asian		0.06 (0.12)		
Black		0.22 (0.11)		
Other		–0.00 (0.12)		
Hyperactivity			–0.12 (0.08)	–0.14 (0.08)
Emotional problems			0.09 (0.05)	0.07 (0.05)
Conduct problems			–0.00 (0.06)	–0.03 (0.06)
Parameter estimates				
Eating disorder			0.16 (0.10)	0.14 (0.10)
Self-harm			0.06 (0.08)	0.05 (0.08)
Autism			–0.33 (0.08)***	–0.35 (0.08)***
Other problems			–0.09 (0.06)	–0.09 (0.06)
SEN			–0.06 (0.09)	–0.11 (0.09)
Low-frequency case characteristics			–0.14 (0.07)*	–0.13 (0.07)
<1-month duration vs. >1 year				–0.33 (0.26)
1- to 5-month duration vs. >1 year				0.30 (0.11)**
6- to 12-month duration vs. >1 year				0.13 (0.08)
Problem duration missing vs. >1 year				0.04 (0.09)
Moderate distress				0.02 (0.07)
Parameter estimates				
High friendships impairment				0.07 (0.09)
Moderate classroom impairment				0.16 (0.07)
High classroom impairment				0.08 (0.07)
Moderate leisure impairment				–0.047 (0.07)
High leisure impairment				0.09 (0.08)
Variance components				
Residual variance	1.42 (0.01)	1.42 (0.01)	1.39 (0.01)	1.39 (0.01)
Service-level variance	0.06 (0.05)	0.07 (0.06)	0.06 (0.05)	0.05 (0.05)

*N* = 3256. Findings in bold indicate that the pattern (in terms of improvement of model fit and significance of the coefficient) was the same as in the multilevel regressions predicting unadjusted treatment outcome (also see Table 2)

*SEN* special educational needs

* *p* < .05

** *p* < .01

*** *p* < .001

Regarding whether service-level variation in treatment outcome was explained by patient-level demographic, case and severity characteristics, given the small amount of service-level variation, these characteristics explained little service-level variation. The amount of service-level variation did not differ substantially between models with and without patient-level characteristics, ranging between 0.05 and 0.07. Findings will be discussed for unadjusted and adjusted treatment outcome below.

### Unadjusted treatment outcome

Adding demographic characteristics in Model 1 improved the model fit, but the ICC increased to 6%; likelihood ratio test: *χ* ^2^(8) = 237.80, *p* < 0.05. Older young people had lower risk of poor outcomes^3^ than younger young people. Black young people had lower risk of poor outcomes than young people with unstated or missing ethnic identifiers. Adding case characteristics in Model 2 improved the model fit but the ICC remained 6%; likelihood ratio test: *χ* ^2^(9) = 38.46, *p* < 0.05. Young people presenting with an eating disorder at the outset of treatment had lower risk of poor outcomes than young people without this characteristic. In contrast, young people presenting with hyperactivity, autism, or infrequent case characteristics at the outset of treatment had greater risk of poor outcomes than young people without these case characteristics. Adding severity characteristics in Model 4 did not improve the model fit; likelihood ratio test: *χ* ^2^(14) = 12.87, *p* > 0.05.

### Adjusted treatment outcome

In contrast to the findings with unadjusted treatment outcome, adding demographic characteristics in Model 1 did not improve the model fit; likelihood ratio test: *χ* ^2^(8) = 14.38, *p* > 05. Similar to the findings with unadjusted treatment outcome, adding case characteristics in Model 2 significantly improved the model fit but the ICC remained 4%; likelihood ratio test: *χ* ^2^(9) = 22.19, *p* < 0.05. Similar to the findings with unadjusted treatment outcome, young people presenting with autism or infrequent characteristics at the outset of treatment had greater risk of poor outcomes than young people without these characteristics. In contrast to the findings with unadjusted treatment outcome, the effects of eating disorder and hyperactivity were not significant. Similar to the findings with unadjusted treatment outcome, adding severity characteristics in Model 4 did not improve the model fit; likelihood ratio test: *χ* ^2^(14) = 23.14, *p* > 0.05.

## Discussion

This research investigated (1) the amount of service-level variation in treatment outcome in child mental health, (2) whether it differed when examining outcomes unadjusted versus adjusted for expected change over time, and (3) which patient-level characteristics were associated with the difference observed between services.

There was a relatively small amount of service-level variation, in line with previous evidence showing therapist-level effects of 6–9% [25]. Amounts of service-level variation did not differ greatly according to unadjusted versus adjusted treatment outcome or across models. Given the small amount of service-level variation, demographic, case and severity characteristics explained little service-level variation. Young people with autism or infrequent case characteristics requiring specialist input had greater risk of poor outcomes using these methods of measuring change.

There were some effects that were significant when examining unadjusted treatment outcome but not when examining adjusted treatment outcome, and future research should examine reasons for the difference in pattern of findings, particularly in terms of co-morbidity, which we were unable to examine to avoid over-fitting the models. For instance, it is possible that the effect of eating disorder on unadjusted treatment outcome was significant because, unlike with adjusted treatment outcome, it did not account for young people’s level of emotional problems at the outset of treatment. Perhaps adjusted treatment outcome was accounting for co-morbid eating and emotional problems, whereas unadjusted treatment outcome was not.

Limitations should be considered when interpreting the findings of the present research. The present research used naturalistic, routinely collected data as opposed to those collected under controlled conditions. Therefore, limitations of confounding variables and selection bias may apply [26] and future research is needed to replicate the findings of the present research, particularly to explore which factors explain the large amount of unexplained variance, such as clinician-level factors [25]. The use of the CORC dataset means that there may be some variation in how data were collected and recorded, as individual services may have collected and coded information differently, which has been noted as a limitation when attempting risk adjustment in physical health settings [27]. Case characteristics occurring with a frequency of <5% were grouped into an ‘infrequent characteristics’ variable to avoid including under-powered groups in the main analysis; however, this resulted in a heterogeneous variable. Although the present study was based on a large national dataset including data from 13 services, findings may not generalise to other services in the UK. There was a relatively small proportion of young people with complete longitudinal data compared to those without, and there were differences between the complete and incomplete samples (see “Methods”). Still, a strength of the present research is that it examined unadjusted treatment outcome and treatment outcome adjusted for expected change had young people not received intervention. Nevertheless, as the method for adjusting treatment outcome was developed in a sample of young people with clinical mental health difficulties [7], it is possible that it may underestimate spontaneous improvement and overestimate effects of treatment in young people with milder difficulties. Future studies should replicate the findings of the present research using larger samples with a heterogeneous range of mental health difficulties.

Notwithstanding the above limitations, the present research provided evidence as to the amount of service-level variation in outcomes in child mental health, whether it differed when examining outcomes unadjusted versus adjusted for expected change over time, and which patient-level characteristics were associated with the difference observed between services. There was 4–5% service-level variation in outcomes. Findings were broadly consistent across unadjusted versus adjusted outcomes. Young people with autism or infrequent case characteristics requiring specialist input had greater risk of poor outcomes. We recommend caution when comparing service-level outcomes regarding child mental health and well-being, especially as there appears to be much more variation between patients than between child mental health services. Methods that account for imprecision in service-level estimates (e.g., funnel plots) are recommended. Comparison of services with high proportions of young people with autism or infrequent characteristics requiring specialist input needs particular caution to adjust for these groups who may be at greater risk of poor outcomes on some measures, as it may appear that these services are performing worse than other services when in fact differences may be attributable to different patient characteristics.

## Footnotes

1 There have been recent calls for child mental health services to be structured according to needs-based groupings but they are currently structured according to four Tiers: Tier 1) non-specialist support for common problems of childhood (e.g., sleeping), Tier 2) specialist support provided in primary care settings (e.g., bereavement), Tier 3) specialist multidisciplinary child and adolescent mental health teams based in local clinics dealing with more complex problems (e.g., autism) representing the majority of services in the present research, and Tier 4) specialised day and inpatient units for patients with more severe mental health problems [28].

2 In the CORC dataset, pseudonymized data are uploaded according to episodes of care. Therefore, it is possible that a young person may have been included under more than one episode of care.

3 Poor outcome is defined as an increase in mental health difficulties from Time 1 to Time 2.
